# An all integer-based spiking neural network with dynamic threshold adaptation

**DOI:** 10.3389/fnins.2024.1449020

**Published:** 2024-12-17

**Authors:** Chenglong Zou, Xiaoxin Cui, Shuo Feng, Guang Chen, Yi Zhong, Zhenhui Dai, Yuan Wang

**Affiliations:** ^1^Peking University Chongqing Research Institute of Big Data, Chongqing, China; ^2^School of Mathematical Science, Peking University, Beijing, China; ^3^School of Integrated Circuits, Peking University, Beijing, China

**Keywords:** spiking neural network, dynamic threshold adaptation, ANN2SNN conversion, network quantization, neuromorphic computing

## Abstract

Spiking Neural Networks (SNNs) are typically regards as the third generation of neural networks due to their inherent event-driven computing capabilities and remarkable energy efficiency. However, training an SNN that possesses fast inference speed and comparable accuracy to modern artificial neural networks (ANNs) remains a considerable challenge. In this article, a sophisticated SNN modeling algorithm incorporating a novel dynamic threshold adaptation mechanism is proposed. It aims to eliminate the spiking synchronization error commonly occurred in many traditional ANN2SNN conversion works. Additionally, all variables in the proposed SNNs, including the membrane potential, threshold and synaptic weights, are quantized to integers, making them highly compatible with hardware implementation. Experimental results indicate that the proposed spiking LeNet and VGG-Net achieve accuracies exceeding 99.45% and 93.15% on the MNIST and CIFAR-10 datasets, respectively, with only 4 and 8 time steps required for simulating one sample. Due to this all integer-based quantization process, the required computational operations are significantly reduced, potentially providing a substantial energy efficiency advantage for numerous edge computing applications.

## 1 Introduction

In recent years, neuromorphic computing based on spiking neural networks (SNNs) (Bouvier et al., 2019) has attracted more and more attention across computer vision (CV), automatic speech recognition (ASR) and natural language processing (NLP) tasks. SNNs are a special type of neural network that operate based on the principles of biological neural systems (Kasabov, 2019). At the functional level, unlike traditional artificial neural networks (ANNs) that use continuous-valued activation functions, SNNs employ spiking neurons that communicate and compute through discrete (0 or 1) spikes. These spikes represent the neural activity and information transmission in the network. At the structure level, SNNs are usually composed of LIF neurons (Andrew, 2003), each of which has a membrane potential which integrates incoming signals from other neurons. When the membrane potential exceeds a certain threshold, the neuron will generate a spike, and then propagated to other connected neurons. This spiking behavior allows SNNs to capture rich spatio-temporal dynamics and feature patterns in data, making them particularly suitable for processing time-varying signals and sequences.

In general, SNNs can offer several advantages compared to traditional ANNs. Firstly, they have a more biologically plausible representation of neural computation, which can lead to better generalization and adaptability to many complex tasks (Schuman et al., 2017). Secondly, SNNs are typically more energy-efficient because they usually communicate through sparse spikes and compute in an event-driven style (Taherkhani et al., 2020). This could significantly reduce the amount of data transfer and computation energy requirements (Bouvier et al., 2019; Schuman et al., 2017).

However, on the one hand, training SNNs remains a big challenge due to their discrete and event-driven characteristics. Traditional gradient-based optimization methods used in ANNs are not directly applicable to SNNs (Taherkhani et al., 2020; Tavanaei et al., 2019). Therefore, researchers have developed specialized learning algorithms and techniques to train SNNs effectively, such as spike-timing-dependent plasticity (STDP) (Neftci et al., 2019), surrogate gradient learning (SGL) (Lee et al., 2019) and ANN2SNN conversion (Dampfhoffer et al., 2023). Direct SGL algorithms originate from the training strategy of the recurrent neural networks (RNN) (Hochreiter and Schmidhuber, 1997) and can be directly applied on some complex network architectures, such as residual neural network (ResNet) (He et al., 2016) with batch normalization (BN) (Ioffe and Szegedy, 2015). However, their performance is limited by the fitness of the gradient approximation function, the training process is usually time or memory-consuming. Local STDP algorithm and its variants are only suitable for shallow learning and couldn't achieve end-to-end training for deep networks. ANN2SNN conversion is a kind of two-stage SNN modeling method, which can convert a decent ANN trained with back-propagation (BP) algorithm (Li et al., 2021) to an equivalent SNN based on the firing rate approximation. However, these converted SNNs usually suffer from uncertain accuracy loss (Bodo et al., 2017) when tested on some larger-scale datasets such as CIFAR-10 (Krizhevsky and Hinton, 2009) or ImageNet (Russakovsky et al., 2015) within fewer time steps.

On the other hand, only when SNNs are deployed on some specialized neuromorphic hardware such as TrueNorth (Esser et al., 2016) and Loihi (Massa et al., 2020) chip, can they really show the advantages of high energy efficiency. This means the low-bit quantization of synaptic weights, firing thresholds and leakage terms of an SNN must be necessary. However, many SNN works (Dampfhoffer et al., 2023; Li et al., 2021; Bodo et al., 2017) only concentrate on the improvement of inference accuracy and speed, but ignore the hardware friendliness of their models. In this article, we present a delicate SNN architecture with dynamic threshold adaptation mechanism, to eliminate the common synchronization errors existed in many other ANN2SNN conversion works. Besides, all SNN parameters including membrane potential and firing threshold can be quantized to integers, which is very friendly for hardware implementation. Experimental results show that the proposed spiking LeNet (Lecun and Bottou, 1998) and VGG-Net (Simonyan and Zisserman, 2014) can obtain more than 99.45% and 93.15% classification accuracy on MNIST (Lecun and Bottou, 1998) and CIFAR-10 (Krizhevsky and Hinton, 2009) datasets with only 4 and 8 time steps, respectively.

The rest of this article is organized as follows. Section II outlines the details of the proposed ANN2SNN conversion algorithm, Section III introduces the parameter quantization and spike input encoding techniques. Section IV provides the experiment results and section V draws the conclusion.

## 2 ANN2SNN conversion

In this section, we would first briefly analyze the two kinds of common conversion loss among many previous ANN2SNN works. And then, a novel dual-threshold spiking approach which is also called dynamic threshold adaptation, is introduced to eliminate these errors from the source.

### 2.1 Conversion loss analysis

**Figure 1:**
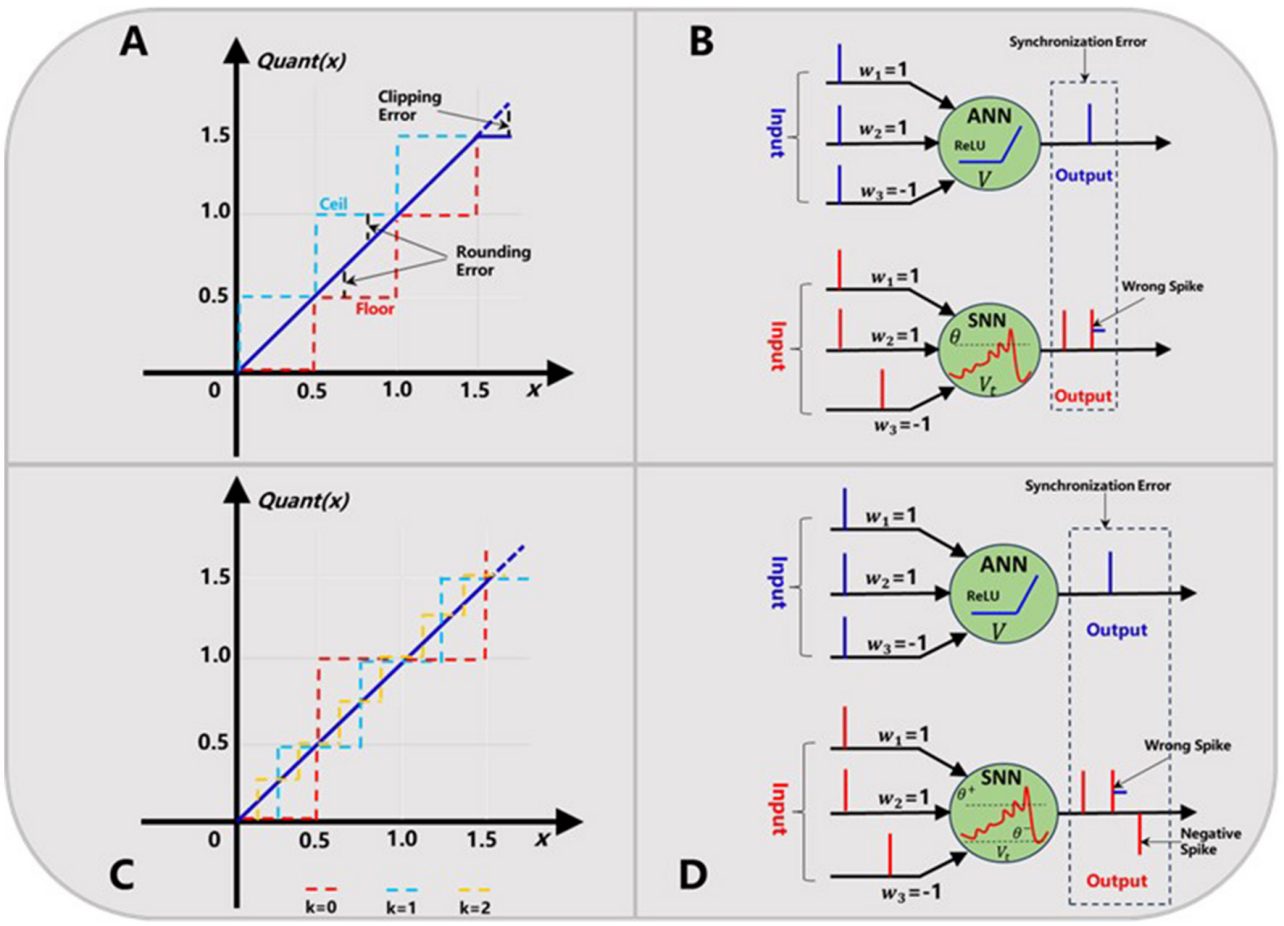
Quantization error **(A)** and synchronization error **(B)** in ANN2SNN conversion. Trainable quantization function **(C)** and dual-threshold spiking mechanism **(D)** for solving the above two problems, respectively.

Conventional ANN2SNN conversion algorithms such as the weight normalization (Bodo et al., 2017; Diehl et al., 2015) and threshold normalization (Xu et al., 2017; Zou et al., 2020) inevitably suffer from some accuracy loss, due to the commonly existed quantization error and synchronization error. As shown in Figure 1A, the quantization error usually includes the rounding error and clipping error. The rounding error can be further divided into the ceiling and flooring error depending on the actual approximation method between the ReLU activation (Glorot et al., 2011) of ANN neuron and the firing rate of SNN neuron. The clipping error occurs because when the upper bound of the ReLU activation function is usually truncated to achieve a fast simulation within fewer time steps (Chen et al., 2022).

Then, the synchronization error is also called sequential error in other work (Chen et al., 2022; Hu et al., 2023), which is caused by the difference of sequential firing mechanism of spikes in SNNs and static activation propagation in ANNs. Usually, the synchronization error is accumulated in higher layers at some earlier time steps, and causes the serious output mismatch between ANNs and their SNN counterparts. For example, as shown in Figure 1B, if we set the firing threshold to 1 and use a threshold subtraction scheme after a spike generation, this SNN neuron will fire for twice. This means a wrong spike will be generated, which is obviously not equivalent with the original ANN neuron outputs. This may be a more fundamental and much tougher problem which needs to be addressed.

Generally, both quantization error and synchronization error would degrade the accuracy performance to some extent in many previous converted SNNs (Diehl et al., 2015; Xu et al., 2017). As far as we know, the quantization error including the rounding error and clipping error can be alleviated by means of some quantization-aware training (QAT) methods (Chen et al., 2022; Hu et al., 2023) and longer simulation time, however the synchronization error serves as an inherent attribute in ANN2SNN conversion algorithms, which is key problem to be dealt with.

### 2.2 Dynamic threshold adaptation

To overcome the effects of the synchronization error, many researchers have tried various useful ways. For example, Bodo et al. (2017) and Sengupta et al. (2019) adopt a conventional method of increasing simulation time steps to cover up the wrongly fired spikes. This method could improve the final accuracy to some extent, but bring longer simulation latency. Zou et al. (2020) and Meng et al. (2022) regularizes the input spikes of the first SNN layer to obtain a more uniform spike sequence representation. However, for much deeper networks, the spiking activities in the middle layers of SNNs are usually very complicated and exactly unpredictable and thus these spikes are almost impossible to be regularized.

In this work, we present a novel dual-threshold spiking approach together with a median quantization constraint to eliminate the two errors described above simultaneously. Firstly, each ReLU output value will be quantized with a hyper-parameter *k* called quantization level as Equation (1). It should be noted this parameter is trainable to minimize the quantization error and determines the quantization precision of ANN outputs as in Figure 1C. Then, we can convert this ANN to a firing-rate based SNN based on the following procedures. As in Equation 2–4, the converted spiking neuron works as the normal LIF behavior (Andrew, 2003), but has two thresholds i.e. θ^+^and θ^−^, which determines if a positive or negative spike will be generated respectively. What's different is that these two thresholds are dynamic along with simulation time step *t* and will be updated synchronously. This dual-threshold spiking mechanism can be further elaborated in Figure 1D, where one negative spike is produced to correct the wrongly fired spike when the membrane potential *V*_*t*_ falls below the negative threshold θ^−^.


(1) $$\begin{array}{l} Quant\left(y\right)=\frac{Round\left(y\times 2^{k}\right)}{2^{k}} \end{array}$$

(2) $$\begin{array}{l} V_{t+1}=\;V_{t}+\sum \left(o_{t}\times w\right) \end{array}$$

(3) $$\begin{array}{l} o_{t+1}=\{\begin{array}{c} 1,\;if\;V_{t+1}\geq \theta_{t}^{+}\\ -1,\;if\;V_{t+1}\;<\theta_{t}^{-}\\ 0,\;else \end{array} \end{array}$$

(4) $$\begin{array}{l} \theta_{t+1}^{+}/\theta_{t+1}^{-}=\{\begin{array}{c} \theta_{t}^{+}/\theta_{t}^{-}+\delta ,\;if\;o_{t+1}=1\\ \theta_{t}^{+}/\theta_{t}^{-}-\delta ,\;if\;o_{t+1}=-1\\ \theta_{t}^{+}/\theta_{t}^{-},\;else \end{array} \end{array}$$

(5) $$\begin{array}{l} \delta =\frac{\frac{1}{2^{k}}\times \left(\sigma^{\prime }+\epsilon \right)}{\gamma } \end{array}$$

(6) $$\begin{array}{l} \theta_{0}^{+}=\frac{\left(\frac{1}{2^{k+1}}-\beta \right)\left(\sigma^{\prime }+\epsilon \right)}{\gamma }+\mu^{\prime } \end{array}$$

(7) $$\begin{array}{l} \theta_{0}^{-}=\theta_{t}^{+}-\delta \; \end{array}$$

Besides, the regulating term of two thresholds i.e. δ, and initial value $\theta_{0}^{+}$/$\theta_{0}^{-}$ in Equation 5–7 are derived from Equation (1) and the BN parameters, just like Zou et al. (2023). It should be noted that this mechanism extends from the previous work (Zou et al., 2023), but features with following key characteristics:

- **More simplified computation and less memory consumption**: The original high-precision shadow membrane potential of each spiking neuron in Zou et al. (2023) is removed in this work. This improvement could significantly reduce the computational complexity, memory burden of SNNs and speed up the calculation process.
- **Higher biological plausibility**: The positive and negative threshold change synchronously, if a positive (+1) or negative spike (−1) is generated at time step *t*, both of the two thresholds will change accordingly to prevent firing again at this level. This working mechanism may be more biologically plausible because of its similarity to the refractory period phenomenon (Kasabov, 2019).

Compared with the negative spiking mechanism proposed in Hu et al. (2023), there are at least following differences: (1) The signed IF neuron in Hu et al. (2023) needs an auxiliary variable to record the number of spikes each neuron has fired at the current time step, while our spiking neurons doesn't require extra variable. (2) The threshold (positive and negative) in Hu et al. (2023) is static along time step, while ours is dynamic and also more biologically plausible.

## 3 Neural network quantization

In this section, we first introduce the input spike encoding algorithm, i.e. the conversion process of the static image pixels to input sequential spikes. Then, an all integer-based parameter quantization method will be presented to build a hardware-friendly SNN model.

### 3.1 Input spike encoding

**Figure 2:**
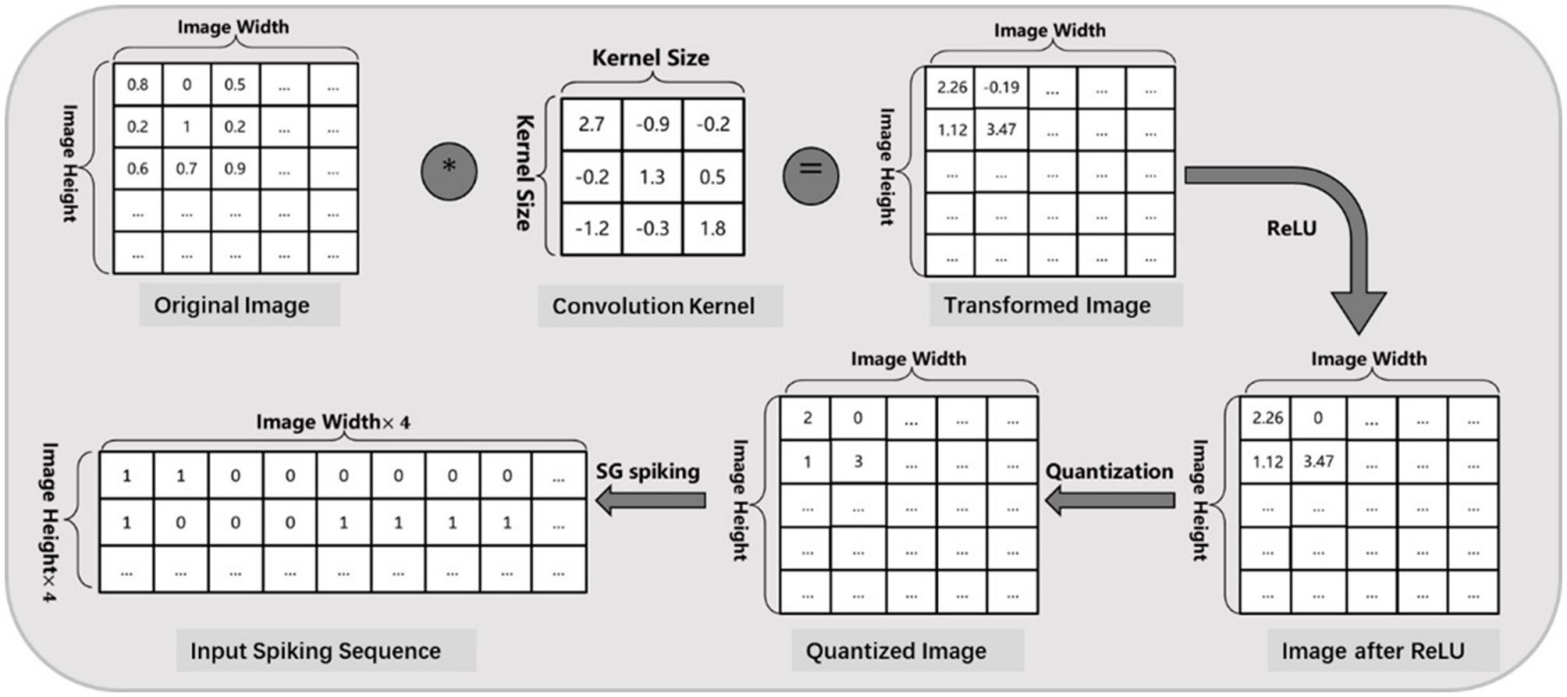
Input image encoding process based on SG spiking algorithm (Zou et al., 2021), where each intensity value will be discretized into a spike sequence within 4 time steps.

In general, lots of static data in nature such as image and text, would be collected and used for ANN training, but must be converted to spiking signals before it can be fed into SNNs during inference. In this article, we adopt a constant scatter-and-gather (SG) encoding algorithm as in Zou et al. (2021) to minimize the information loss of the input layer between ANN and SNN models. As shown in Figure 2, any intensity value will be processed through a normal convolutional layer and then discretized into a spike sequence with only 4 time steps. This skill could further accelerate SNN simulation (smaller time window) and decrease the total computation cost. It should be noted that the weight parameters of this encoding layer are trainable but not quantized, and these spiking neurons only act as the original LIF behavior (Andrew, 2003) without negative spiking mechanism.

### 3.2 Parameter quantization

Ultimately, the ultra-high energy efficiency strength of SNNs comes from two important features: **(1) low parameter precision** and **(2) sparsity calculation**. This means that only when SNNs are deployed on some dedicated neuromorphic hardware, can they reach their real low-power potential. However, many SNN works (Taherkhani et al., 2020; Neftci et al., 2019; Dampfhoffer et al., 2023) mainly concentrate on the model accuracy improvements but omit their hardware friendliness. In this article, we present an all integer-based parameter quantization approach for both synaptic weights and firing thresholds, which would greatly facilitate the hardware implementation for the proposed SNN models.

#### 3.2.1 Weight quantization

We adopt a lightweight ternary quantization scheme as in Liu et al. (2023) for the synaptic weights in convolutional and fully connected layers. As in Equation 8, the floating-point weights will be quantized to only 0,1 or −1 depending on a specific threshold = 0.7 × |*W^f^*|. Besides, we adopt a straight-through estimator (STE) function (Bengio et al., 2013) as in Equation 9 to pass the gradients backwards through the networks. Because both inputs and outputs of spiking neurons are in the form of spike sequence (0,1 or −1), the multiplication-addition (MAC) calculations in these layers could be implemented by only bit-operation such as *XNOR-popcount* in Courbariaux et al. (2016). As a typical quantization aware training (QAT) approach, the ternary weights in ANNs are trainable and will be unchanged when converted into the SNN version. This quantization process for weight would significantly increase the simplicity of massive spike integration calculation.


(8) $$\begin{array}{l} W^{T}=\{\begin{array}{cc} 1, & if\;W^{F}>0.7\times \left|W^{F}\right|\\ 0, & if\;|W^{F}|\leq 0.7\times \left|W^{F}\right|\\ -1, & if\;W^{F}<-0.7\times \left|W^{F}\right| \end{array} \end{array}$$

(9) $$\begin{array}{l} \frac{\partial L}{\partial W^{F}}=\frac{\partial L}{\partial W^{T}}\times \frac{\partial W^{T}}{\partial W^{F}}\approx \{\begin{array}{c} \frac{\partial L}{\partial W^{T}}\;if\;{|W}^{F}|\leq 1\\ \;0\;if\;{|W}^{F}|>1\; \end{array} \end{array}$$

#### 3.2.2 Threshold quantization

As shown in Equations 5–7, each SNN neuron has two firing thresholds $\theta_{0}^{+}$, $\theta_{0}^{-}$ and one regulating term δ. These parameters consist of the mean μ, variance σ, scaling γ and shift β terms in BN **(Ioffe and Szegedy**, **2015****)** layers, which are originally all floating-point numbers. Here, we firstly scale the weights **W**^**T**^ and firing thresholds $\theta_{0}^{+}$, $\theta_{0}^{-}$ by 10,000 × and then use a ***Round*** function as in Equation 10 to quantize them into all integers for hardware-equivalent mapping. It should be mentioned that the quantization test for firing threshold is very rare in many previous SNN works. Based on our multiple experimental tries, we found these parameters are not sensitive to the choice of quantization function including ***Floor***, ***Round*** and ***Ceil***, etc. We will discuss the effects of quantization for these parameters in the next section.


(10) $$\begin{array}{l} Quant\left(\theta_{0}^{+}/\theta_{0}^{-}/\delta \right)=Round\left(\theta_{0}^{+}/\theta_{0}^{-}/\delta \times 10000\right) \end{array}$$

## 4 Result and discussion

In this section, we firstly test the proposed ANN2SNN conversion method on both MNIST and CIFAR-10 dataset and present the experimental results including the inference accuracy and speed, together compared with several state-of-the-art works of similar network sizes. Then, we carry out an estimation of spiking sparsity and synaptic operations (SOPs) of the proposed spiking models, to verify their great energy efficiency. All models are trained using standard ADAM (Krizhevsky and Hinton, 2009) rule with an initial learning rate set to 0.01 and we don't use any data augmentation other than a standard random image flipping and cropping for CIFAR-10. It should be noted our ANN2SNN conversion method can be compatible with many other optimization and regularization skills (Cheng et al., 2017) and some advanced architectures like ResNet (Li et al., 2022) for further better performances. More details are available from our online Python implementation code (https://github.com/edwardzcl/All_INT_SNN).

### 4.1 Experiments on MNIST

MNIST handwritten digit dataset (Lecun and Bottou, 1998) has been widely applied in image classification field, which was collected from the postal codes including a training set of 60,000 examples, and a test set of 10,000 examples. Each example is an individual 28 × 28-pixel grayscale image, labeled 0~9. For this task, we adopt a classical 6-layer LeNet architecture (Lecun and Bottou, 1998), i.e., 32*C*5-2*P*2-64*C*5-2*P*2-1000*FC*-10*FC* where *C, P* and *FC* denoted convolution, pooling and fully connected layer respectively and 32*C*5 represents a convolution layer with 32 output channels and 5 × 5 kernel size. It should be noted that the input layer and output layer are not quantized and used for the spike encoding and loss calculation respectively. We firstly conduct an ablation study on the quantization level *k* based on a choice of {0,1,2} as well as the weight, threshold quantization process. These parameter configurations determine the quantization precision of an SNN model and how many spikes each neuron will fire, which is related to total power consumption and latency in network. As shown in Table 1, all predictions stabilize quickly, and there is only little impact on the final stable accuracies for different configurations, except for the final stable time steps. The proposed quantization has almost no effect on model accuracy, but higher quantization levels will bring longer simulation time. To our surprise, the SNN with *k*=2 can achieve an accuracy that is comparable to its full-precision ANN counterpart. For a comparison, we summarize our results (for *k*=0) and other state-of-the-art works in Table 2. It shows that the spiking LeNet with both weight and threshold quantization is nearly lossless with its ANN counterpart (smallest accuracy loss), and can even achieve great accuracy and speed advantages among many other works with full-precision parameters.

**Table 1:** Classification accuracy for LeNet of different configurations on MNIST.

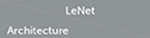		**Weight quantization**	**Threshold quantization**	**Final accuracy**	**Final time step**
ANN		×	-	**99.45%**	-
		✓		99.42%	
SNN	*k* = 0	×	×	99.44%	7
		✓	×	99.40%	**6**
		×	✓	99.41%	**6**
		✓	✓	99.42%	**6**
	*k* = 1	×	×	99.41%	13
		✓	×	99.40%	11
		×	✓	99.45%	12
		✓	✓	99.43%	10
	*k* = 2	×	×	99.49%	25
		✓	×	99.42%	22
		×	✓	99.43%	24
		✓	✓	**99.45%**	21

The **bold** number represents the best in the column.

**Table 2:** Comparison for the proposed spiking LeNet on MNIST with other works.

**References**	**Network structure**	**ANN (full precision)**	**SNN (all integer for ours and high precision for others)**						**Accuracy loss**
			**T** = **2**	**T** = **4**	**T** = **8**	**T** = **16**	**T** = **32**	**T** **≥64**	
Zou et al. (2020)	LeNet	99.29%	-	99.29%	-	-	-	-	**0.00%**
Bu et al. (2022)	LeNet	99.07%	-	-	-	-	-	99.09%	−0.02%
Tong et al. (2022)	LeNet	99.19%	-	-	-	99.17%	-	-	0.02%
Howard et al. (2017)	LeNet	99.21%	-	-	99.19%	-	-	-	0.02%
Diehl et al. (2015)	LeNet	99.14%	9.80%	9.80%	9.84%	93.73%	98.93%	98.99%	0.15%
Bodo et al. (2017)	LeNet	99.44%	12.98%	88.03%	98.17%	99.25%	**99.44%**	**99.44%**	**0.00%**
Zou et al. (2023)	LeNet	99.29%	**99.18%**	99.31%	99.29%	99.29%	99.29%	99.29%	**0.00%**
***k*** **=** **0 (This work)**	LeNet	**99.45%**	99.09%	**99.45%**	**99.42%**	**99.42%**	99.42%	99.42%	0.03%

The **bold** number represents the best in the column.

### 4.2 Experiments on CIFAR-10

CIFAR-10 (Krizhevsky and Hinton, 2009) is regarded as a more challenging real image classification dataset, which consists of total 60000 color images with 32 × 32 pixels. This dataset is divided into 50000 training images and 10000 test images with 10 classes. For this task, a VGG-Net (Simonyan and Zisserman, 2014) variant with 11 layers (96*C*3-256*C*3-2*P*2-384*C*3-2*P*2-384*C*3-256*C*3-2*P*2-1024*C*3-1024FC-10*FC*) is designed. No extra data augmentation technique is used other than standard random image flipping and cropping for training. Test evaluation is based solely on central 24 × 24 crop from test set.

Similarly, we give the ablation study results of different quantization configurations in Table 3 and compare the performance results with other works in Table 4. It also shows that higher quantization levels could bring slightly better accuracy while longer simulation time steps. Besides, the reported inference accuracy and speed of spiking VGG-Net in Table 4 indicates that our proposed conversion and quantization method can still maintain excellent performance (accuracy vs. speed) with the smallest accuracy loss for deeper VGG-Net with more than 10 layers and complex BN operations (Ioffe and Szegedy, 2015). Compared with many other high-precision SNN works, our proposed spiking models are all integer-based and show strong potential for direct implementation on some dedicated hardware.

**Table 3:** Classification accuracy for VGG-Net of different configurations on CIFAR-10.

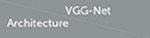		**Weight quantization**	**Threshold quantization**	**Final accuracy**	**Final time step**
ANN		×	-	**93.32%**	-
		✓		93.29%	
SNN	*k* = 0	×	×	93.26%	13
		✓	×	93.19%	11
		×	✓	93.12%	12
		✓	✓	93.11%	**10**
	*k* = 1	×	×	93.22%	18
		✓	×	93.19%	16
		×	✓	93.20%	17
		✓	✓	93.15%	15
	*k* = 2	×	×	93.27%	32
		✓	×	93.21%	30
		×	✓	93.29%	32
		✓	✓	93.21%	29

The **bold** number represents the best in the column.

**Table 4:** Comparison for the proposed spiking VGG-Net on CIFAR-10 with other works.

**References**	**Network structure**	**ANN (full precision)**	**SNN (all integer for ours and high precision for others)**						**Accuracy loss**
			**T** = **4**	**T** = **8**	**T** = **16**	**T** = **32**	**T** = **64**	**T** **≥** **128**	
Li et al. (2021)	VGG-16	93.51%	-	-	-	91.82%	92.57%	93.21%	0.30%
Meng et al. (2022)	ResNet-20	93.18%	-	-	89.17%	91.91%	92.68%	92.92%	0.26%
Zou et al. (2023)	VGG-11	92.66%	91.29%	92.88%	92.65%	92.64%	92.64%	92.64%	0.02%
Deng and Gu (2021)	VGG-16	**95.60%**	-	91.41%	**93.64%**	**94.81%**	-	-	0.79%
Gao et al. (2023)	VGG-16	92.09%	-	-	92.29%	92.29%	92.22%	92.24%	−0.15%
Bu et al. (2022)	VGG-16	94.02%	-	80.28%	90.35%	93.10%	**93.32%**	**93.68%**	0.34%
Yousefzadeh et al. (2019)	ResNet-20	92.74%	-	66.24%	87.22%	91.88%	92.57%	92.73%	0.01%
Tong et al. (2022)	ResNet-20	91.77%	83.75%	89.55%	91.62%	92.24%	92.35%	-	**−0.58%**
***k*** **=** **0 (This work)**	VGG-11	93.32%	**92.05%**	**93.15%**	93.11%	93.11%	93.11%	93.11%	0.21%

The **bold** number represents the best in the column.

### 4.3 Experiments on CIFAR-100 and ImageNet

CIFAR-100 (Krizhevsky and Hinton, 2009) is just like the CIFAR-10 but more challenging. It has 100 classes containing 600 images each. There are 500 training images and 100 testing images per class. ImageNet (Russakovsky et al., 2015) is a much larger dataset, which consists of more than one million image samples and falls into 1000 categories. To verify the effect of our conversion algorithm on these two datasets, we adopt the VGG-11 (the same as the network for CIFAR-10) and a 29-layer MobileNet-V1 (43) for experiment running, respectively. Similarly, we do not use any other optimization techniques for training and the test evaluation is based solely on central crop from test set. It should be noted we train MobileNet-V1 on ImageNet dataset for only 60 epochs, because it needs quite long simulation time and vast parallel computing resources. The experimental results on these two large-scale datasets are summarized in Tables 5, 6, and some comparison data of (Gao et al., 2023; Bu et al., 2022) are collected from self-implementation results (Li et al., 2021). It can be seen that the accuracies of both the proposed spiking VGG-Net and MobileNet could achieve much faster convergence along early time steps, when compared with other works respectively. This phenomenon may be attributed to our good solution of synchronization error which is discussed in Section 2.1. The final accuracy is slightly damaged because our ANN counterparts are trained using some basic optimization techniques and fewer epochs.

**Table 5:** Comparison for the proposed spiking VGG-Net on CIFAR-100 with other works.

**References**	**Network structure**	**ANN (full precision)**	**SNN (all integer for ours and high precision for others)**						**Accuracy loss**
			**T** = **4**	**T** = **8**	**T** = **16**	**T** = **32**	**T** = **64**	**T** **≥128**	
Li et al. (2021)	VGG-16	70.21%	-	-	-	64.53%	67.14%	68.99%	1.22%
Li et al. (2021)	MobileNet	73.23%	-	-	-	40.06%	62.81%	69.41%	3.82%
Li et al. (2021)	ResNet-20	68.40%	-	-	-	65.14%	67.63%	68.28%	0.12%
Meng et al. (2022)	ResNet-20	70.15%	-	-	59.61%	66.24%	69.14%	69.99%	0.16%
Gao et al. (2023)	ResNet-20	**77.16%**	-	-	**-**	51.27%	70.12%	**75.81%**	1.35%
Gao et al. (2023)	VGG-16	77.89%	-	-	-	7.64%	21.84%	55.04%	22.85%
Gao et al. (2023)	MobileNet	73.23%	-	-	-	1.28%	4.88%	39.39%	33.84%
Bu et al. (2022)	VGG-16	72.47%	-	58.58%	66.32%	**71.71%**	**72.68%**	72.85%	−0.38%
Yousefzadeh et al. (2019)	ResNet-20	70.43%	-	23.09%	52.34%	67.18%	69.96%	70.51%	−0.08%
[42]	ResNet-20	69.94%	34.14%	55.37%	**67.33%**	69.82%	70.49%	-	**−0.55%**
***k*** **=** **0 (This work)**	VGG-11	67.40%	**54.27%**	**60.85%**	64.42%	65.21%	65.21%	65.21%	2.19%

The **bold** number represents the best in the column.

**Table 6:** Comparison for the proposed spiking MobileNet on ImageNet with other works.

**References**	**Network structure**	**ANN (full precision)**	**SNN (all integer for ours and high precision for others)**						**Accuracy loss**
			**T** = **16**	**T** = **32**	**T** = **64**	**T** = **128**	**T** = **256**	**T** **≥512**	
Li et al. (2021)	MobileNet	73.40%	-	37.43%	56.26%	65.40%	69.02%	72.38%	1.02%
Li et al. (2021)	ResNet-34	**75.66%**	-	50.21%	63.66%	68.89%	72.12%	**75.44%**	0.22%
Li et al. (2021)	VGG-16	75.36%	-	24.88%	56.77%	**70.49%**	**73.66%**	75.15%	0.21%
Bodo et al. (2017)	VGG-16	63.89%	-	-	-	-	-	49.61%	14.28%
Sengupta et al. (2019)	ResNet-34	70.69%	-	-	-	-	-	65.47%	5.22%
Gao et al. (2023)	ResNet-34	70.95%	-	33.01%	59.52%	67.54%	70.06%	70.98%	**−0.03%**
Gao et al. (2023)	VGG-16	72.40%	-	54.92%	**66.51%**	69.94%	71.35%	72.09%	0.31%
***k*** **=** **0 This work**	MobileNet	70.60%	**47.32%**	**56.15%**	61.89%	64.48%	65.21%	65.21%	5.39%

The **bold** number represents the best in the column.

### 4.4 Energy efficiency

**Figure 3:**
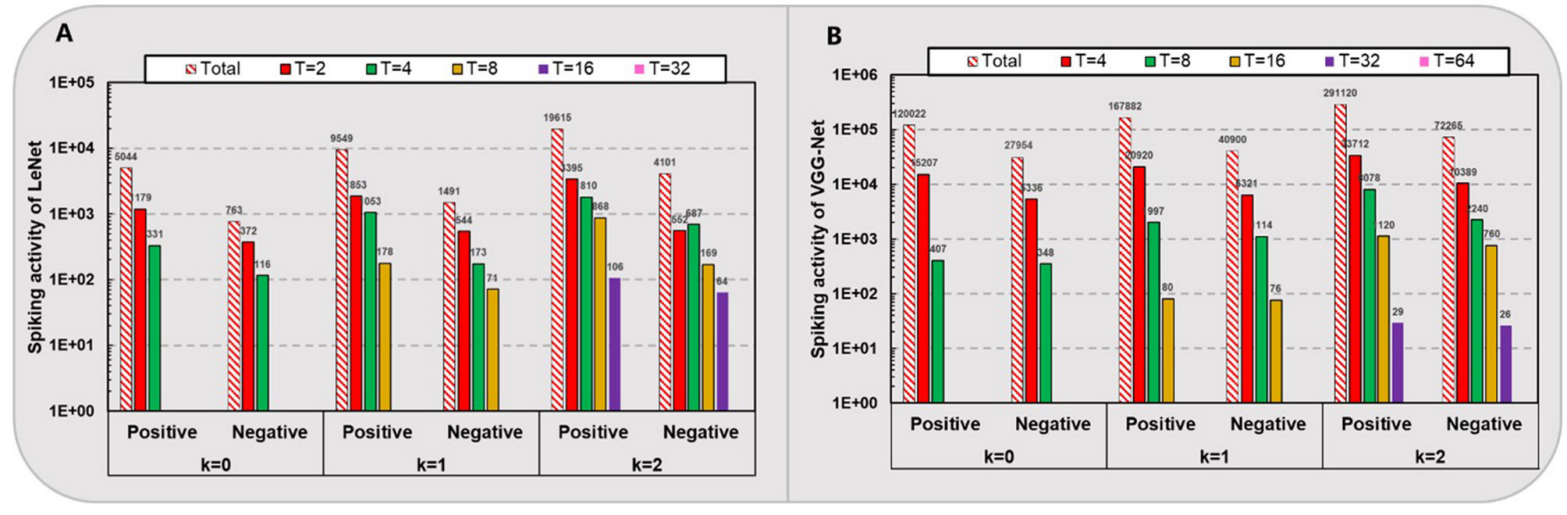
Spiking activity of LeNet **(A)** on MNIST and VGG-Net **(B)** on CIFAR-10.

As shown in Figure 3, we count the average amount of positive and negative spikes for one sample simulation of the spiking LeNet (total 10,728 neurons) on MNIST and VGG-Net (total 280,832 neurons) on CIFAR-10 except for the first input layer and last classification layer. It can be seen that for networks with higher quantization levels, higher spike activities occur while the negative/positive ratio slightly increases. For example, as quantization level *k* varies from 0 to 2, the spike amount on CIFAR-10 for one sample simulation increases from 147,976 to 363,385 (averagely), and the negative/positive ratio of spikes increases from 0.23 to 0.25 (nearly). Overall, there are only about 0.5, 0.74, and 1.3 spikes per neuron with respective *k*ε{0,1,2}. In contrast, the negative/positive ratio of spikes in spiking LeNet (nearly 0.15–0.2) is relatively smaller than VGG-Net (nearly 0.23 to 0.25), which means the negative spikes play a key role in deeper networks with higher quantization levels.

**Figure 4:**
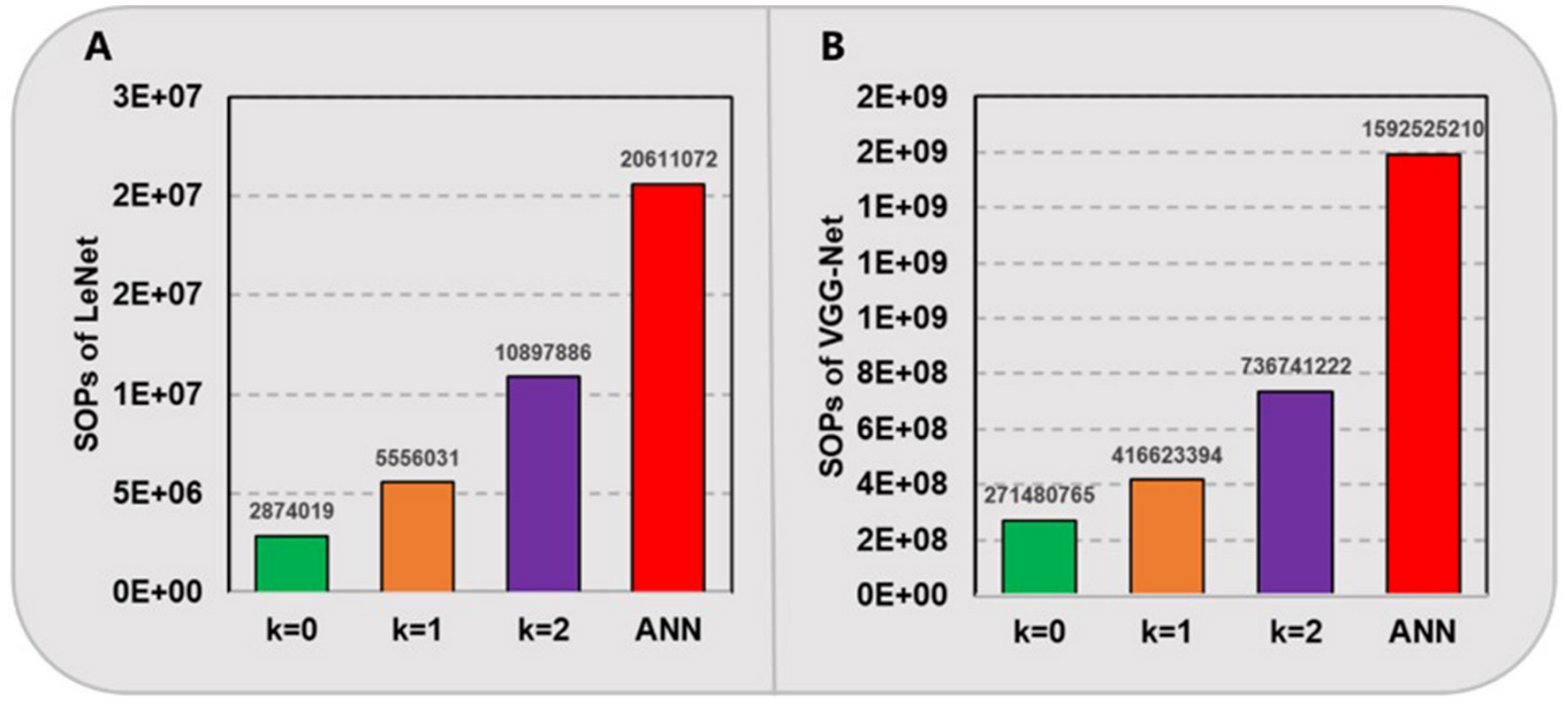
Computational operations (SOPs) of LeNet **(A)** on MNIST and VGG-Net **(B)** on CIFAR-10.

Furthermore, we compare the amount of needed computational operations in above spiking models and their ANN counterparts in Figure 4. For our proposed SNNs with ternary synaptic weights and integer thresholds, there is no need for any high-precision multiplication, only a low-bit SOP, i.e., addition is required when there is a pre-synaptic spike coming. In contrast, for ANNs running on traditional CPUs or GPUs, massive matrix MAC will be performed. Here, we hypothesize that a high-precision MAC is equivalent to 4 low-bit SOPs. In fact, the power and area cost of a floating-point multiplication are always much more expensive than that of several integer-based additions in most of hardware systems (Hu et al., 2023; Courbariaux et al., 2016; Howard et al., 2017). As shown in Figure 4, it can be seen that our proposed SNNs with quantization level *k*ε{0,1,2} consume nearly 7.2, 3.7, and 1.9 times fewer computational operations for LeNet and 5.9, 3.8, and 2.2 times fewer for VGG-Net compared to their ANN counterparts, respectively. These results prove that the converted SNNs can achieve much higher energy efficiency than ANNs, while maintaining comparable accuracy.

Furthermore, because our proposed spiking models run with 0 or ±1 weights and spikes, integer threshold and leakage variables, these integer-based operations could be replaced by the efficient bit-operation such as *XNOR-popcount*, which is introduced in the binary neural networks (BNNs) (Courbariaux et al., 2016) and ternary neural networks (TNNs) (Liu et al., 2023). Even though the computing cost and latency of SNNs may be greater than these two kinds of special ANN-domain models (Tavanaei et al., 2019), the high-accuracy and spatio-temporal processing abilities on some more complex applications still make them the first choice. Of course, a more fair or in-depth comparison between BNNs/TNNs and SNNs may be a perennial topic and will be considered in the future works.

## 5 Conclusion

In this work, we introduce a novel dynamic threshold adaptation technique into traditional ANN2SNN conversion process to eliminate common spike approximation error, and further present an all integer-based quantization method to obtain a lightweight and hardware-friendly SNN model. Experimental results show that the proposed spiking LeNet and VGG-Net can obtain more than 99.45% and 93.15% accuracy on MNIST and CIFAR-10 dataset with only 4 and 8 time steps, respectively. Besides, the captured spiking activity and computational operations in SNNs indicate that our proposed spiking models can achieve much higher energy efficiency with comparable accuracy than their ANN counterparts. Finally, our future works will concentrate on the conversion and quantization methods for some special architecture, such as ResNet, RNN and transformer-based models. More importantly, try to map these models onto some dedicated neuromorphic hardware is more rewarding, this will bring a real running performance improvement for some edge computing applications.

## Data Availability

The original contributions presented in the study are included in the article/supplementary material, further inquiries can be directed to the corresponding authors.
